# Supplementary material for: The Identification of Novel Therapeutic Biomarkers in Rheumatoid Arthritis: A Combined Bioinformatics and Integrated Multi-Omics Approach
Source: Int J Mol Sci. 2025 Mar 19;26(6):2757. doi: 10.3390/ijms26062757 (PMC11943070; doi:10.3390/ijms26062757)
Supplement: Supplementary file 1 [file ijms-26-02757-s001.zip › Supplementary Table S1.pdf]

Table S1: List of KEGG pathways linked with common proteins, boxes highlighted with green color indicates pathways reported to be associated with RA Pathologies with references in corresponding next column, while red boxes indicate no association of pathway was found with RA development

Gene	KEGG Pathways	Reference to Literature
FYN	Sphingolipid signaling pathway	Maceyka, Michael, and Sarah Spiegel. "Sphingolipid metabolites in inflammatory disease." *Nature* 510.7503 (2014): 58-67.
	Phospholipase D signaling pathway	Yoo, Hyun Jung, Won Chan Hwang, and Do Sik Min. "Targeting of phospholipase d1 ameliorates collagen-induced arthritis via modulation of treg and Th17 cell imbalance and suppression of osteoclastogenesis." *International journal of molecular sciences* 21.9 (2020): 3230.
	Axon guidance	Chen, Zhijian, et al. "Expressions of Peptidoglycan Recognition Protein 1, Neuron Towards Axon Guidance Factor-1 and miR-142-3p and Their Correlations in Patients with Rheumatoid Arthritis." *International Journal of General Medicine* (2023): 3457-3464.
	Osteoclast differentiation	Niu, Qing, et al. "Regulation of differentiation and generation of osteoclasts in rheumatoid arthritis." *Frontiers in Immunology* 13 (2022): 1034050.
	Focal adhesion	Shahrara, Shiva, et al. "Differential expression of the FAK family kinases in rheumatoid arthritis and osteoarthritis synovial tissues." *Arthritis research & therapy* 9 (2007): 1-10.
	Adherens junction	Beckmann, Denise, et al. "Lasp1 regulates adherens junction dynamics and fibroblast transformation in destructive arthritis." *Nature Communications* 12.1 (2021): 3624.
	Platelet activation	Harifi, Ghita, and Jean Sibilia. "Pathogenic role of platelets in rheumatoid arthritis and systemic autoimmune diseases: perspectives and therapeutic aspects." *Saudi medical journal* 37.4 (2016): 354.
	Natural killer cell mediated cytotoxicity	Shegarfi, Hamid, Fatemeh Naddafi, and Abbas Mirshafiey. "Natural killer cells and their role in rheumatoid arthritis: friend or foe?." *The Scientific World Journal* 2012.1 (2012): 491974.
	T cell receptor signaling pathway	Weyand, Cornelia M., and Jörg J. Goronzy. "HLA polymorphisms and T cells in rheumatoid arthritis." *International reviews of immunology* 18.1-2 (1999): 37-59.
	Fc epsilon RI signaling pathway	Xu, Fan, et al. "Detection of common pathogenesis of rheumatoid arthritis and atherosclerosis via microarray data analysis." *Heliyon* 10.8 (2024).
	Cholinergic synapse	
	Prion disease	
	Pathogenic Escherichia coli infection	
	Viral myocarditis	
TBL1X	Sphingolipid signaling pathway	Maceyka, Michael, and Sarah Spiegel. "Sphingolipid metabolites in inflammatory disease." *Nature* 510.7503 (2014): 58-67.

**PCYT1B**	Phosphonate and phosphinate metabolism	
	Choline metabolism in cancer	
	Metabolic pathways	Xu, Lingxia, et al. "Metabolomics in rheumatoid arthritis: Advances and review." *Frontiers in immunology* 13 (2022): 961708.
	Glycerophospholipid metabolism	Su, Jiang, et al. "Glycerophospholipid metabolism is involved in rheumatoid arthritis pathogenesis by regulating the IL-6/JAK signaling pathway." *Biochemical and Biophysical Research Communications* 600 (2022): 130-135.
**PTPN13**	Apoptosis	Baier, Anja, et al. "Apoptosis in rheumatoid arthritis." *Current opinion in rheumatology* 15.3 (2003): 274-279.
**DOCK4**	Rap1 signaling pathway	Remans, P. H. J., et al. "Deregulated Ras and Rap1 signaling in rheumatoid arthritis T cells leads to persistent production of free radicals." *Arthritis Research & Therapy* 4 (2002): 1-38.
**F3**	AGE-RAGE signaling pathway in diabetic complications	
	Complement and coagulation cascades	Okroj, Marcin, et al. "Rheumatoid arthritis and the complement system." *Annals of medicine* 39.7 (2007): 517-530.
**UGP2**	Pentose and glucuronate interconversions	Zheng, Tianyao, et al. "Metabolomic analysis of biochemical changes in the serum and urine of Freund's adjuvant-induced arthritis in rats after treatment with silkworm excrement." *Molecules* 23.6 (2018): 1490.
	Galactose metabolism	
	Starch and sucrose metabolism	Xu, Delai, et al. "LC-MS-based rheumatoid arthritis serum metabolomics reveals the role of deoxyinosine in attenuating collagen-induced arthritis in mice." *Heliyon* 10.10 (2024).
	Amino sugar and nucleotide sugar metabolism	Xu, Delai, et al. "LC-MS-based rheumatoid arthritis serum metabolomics reveals the role of deoxyinosine in attenuating collagen-induced arthritis in mice." *Heliyon* 10.10 (2024).
	Metabolic pathways	Xu, Lingxia, et al. "Metabolomics in rheumatoid arthritis: Advances and review." *Frontiers in immunology* 13 (2022): 961708.
	Biosynthesis of cofactors	
	Biosynthesis of nucleotide sugars	
**CALM1**	Ras signaling pathway	Sadeghi Shaker, Mina, et al. "Ras family signaling pathway in immunopathogenesis of inflammatory rheumatic diseases." *Frontiers in Immunology* 14 (2023): 1151246.
	Rap1 signaling pathway	Remans, P. H. J., et al. "Deregulated Ras and Rap1 signaling in rheumatoid arthritis T cells leads to persistent production of free radicals." *Arthritis Research & Therapy* 4 (2002): 1-38.
	Calcium signaling pathway	Wong, Vincent Kam Wai, et al. "Ca2+ signalling plays a role in celastrol-mediated suppression of synovial fibroblasts of rheumatoid arthritis

		patients and experimental arthritis in rats." *British journal of pharmacology* 176.16 (2019): 2922-2944.
	cGMP-PKG signaling pathway	Li, D. Y., et al. "Targeting the nitric oxide/cGMP signaling pathway to treat chronic pain. Neural Regen Res. 2023; 18 (5): 996–1003."
	cAMP signaling pathway	Shu, J., Zhang, F., Zhang, L., & Wei, W. (2017). G protein coupled receptors signaling pathways implicate in inflammatory and immune response of rheumatoid arthritis. *Inflammation research*, *66*, 379-387.
	Phosphatidylinositol signaling system	
	Oocyte meiosis	
	Cellular senescence	
	Adrenergic signaling in cardiomyocytes	
	Vascular smooth muscle contraction	
	Apelin signaling pathway	Chang, Ting-Kuo, et al. "Apelin promotes endothelial progenitor cell angiogenesis in rheumatoid arthritis disease via the miR-525-5p/angiopoietin-1 pathway." *Frontiers in Immunology* 12 (2021): 737990.
	C-type lectin receptor signaling pathway	Takata, Ken, et al. "Elevated macrophage-inducible C-type lectin expression in the synovial tissue of patients with rheumatoid arthritis." *Central European Journal of Immunology* 46.4 (2021): 470-473.
	Circadian entrainment	
	Long-term potentiation	
	Neurotrophin signaling pathway	Lai, Ning-Sheng, et al. "Increased serum levels of brain-derived neurotrophic factor contribute to inflammatory responses in patients with rheumatoid arthritis." *International Journal of Molecular Sciences* 22.4 (2021): 1841.
	Dopaminergic synapse	
	Olfactory transduction	
	Phototransduction	
	Inflammatory mediator regulation of TRP channels	Niu, Mengwen, et al. "The transient receptor potential channels in rheumatoid arthritis: Need to pay more attention." *Frontiers in Immunology* 14 (2023): 1127277.
	Insulin signaling pathway	Tripolino, Cesare, et al. "Insulin signaling in arthritis." Frontiers in immunology 12 (2021): 672519.
	GnRH signaling pathway	Barabás, Klaudia, Edina Szabó-Meleg, and István M. Ábrahám. "Effect of inflammation on female gonadotropin-releasing hormone (GnRH) neurons: mechanisms and consequences." *International journal of molecular sciences* 21.2 (2020): 529.

	Estrogen signaling pathway	Chakraborty, Debolina, et al. "Estrogen-mediated differential protein regulation and signal transduction in rheumatoid arthritis." *Journal of Molecular Endocrinology* 69.1 (2022): R25-R43.
	Melanogenesis	
	Oxytocin signaling pathway	Liang, Guo-Cheng, et al. "Analysis of the composition and anti-rheumatoid arthritis mechanism of Qintengtongbi decoction based on network pharmacology." *Natural Product Communications* 16.9 (2021): 1934578X211041421.
	Glucagon signaling pathway	
	Renin secretion	Moreira, Fernanda Rocha Chaves, et al. "The role of renin angiotensin system in the pathophysiology of rheumatoid arthritis." *Molecular Biology Reports* 48.9 (2021): 6619-6629.
	Aldosterone synthesis and secretion	Bader, Michael. "Renin–Angiotensin–Aldosterone System." *Encyclopedia of Molecular Pharmacology*. Cham: Springer International Publishing, 2022. 1353-1358.
	Salivary secretion	Nagler, R. M., et al. "Salivary gland involvement in rheumatoid arthritis and its relationship to induced oxidative stress." *Rheumatology* 42.10 (2003): 1234-1241.
	Gastric acid secretion	Henriksson, K., et al. "Gastrin, gastric acid secretion, and gastric microflora in patients with rheumatoid arthritis." *Annals of the rheumatic diseases* 45.6 (1986): 475-483.
	Alzheimer disease	
	Parkinson disease	
	Pathways of neurodegeneration - multiple diseases	
	Amphetamine addiction	
	Alcoholism	
	Pertussis	
	Tuberculosis	
	Human cytomegalovirus infection	
	Kaposi sarcoma-associated herpesvirus infection	
	Human immunodeficiency virus 1 infection	
	Pathways in cancer	
	Glioma	
	Lipid and atherosclerosis	Cavagna, Lorenzo, et al. "Atherosclerosis and rheumatoid arthritis: more than a simple association." *Mediators of inflammation* 2012.1 (2012): 147354.

	Fluid shear stress and atherosclerosis	Cavagna, Lorenzo, et al. "Atherosclerosis and rheumatoid arthritis: more than a simple association." *Mediators of inflammation* 2012.1 (2012): 147354.
**COX19**	Thermogenesis	Mao, Xia, et al. "A promising drug combination of mangiferin and glycyrrhizic acid ameliorates disease severity of rheumatoid arthritis by reversing the disturbance of thermogenesis and energy metabolism." *Phytomedicine* 104 (2022): 154216.
**MAGI1**	Rap1 signaling pathway	Remans, P. H. J., et al. "Deregulated Ras and Rap1 signaling in rheumatoid arthritis T cells leads to persistent production of free radicals." *Arthritis Research & Therapy* 4 (2002): 1-38.
	PI3K-Akt signaling pathway	Ba, Xin, et al. "WTD attenuating rheumatoid arthritis via suppressing angiogenesis and modulating the PI3K/AKT/mTOR/HIF-1α pathway." *Frontiers in Pharmacology* 12 (2021): 696802.
	Tight junction	Mucientes, A., et al. "AB0727 TIGHT JUNCTION PROTEINS IN RHEUMATOID ARTHRITIS: ETIOLOGY AND BIOMARKERS." (2024): 1654-1655.
	Human papillomavirus infection	
**SNX3**	Endocytosis	Li, Zhan-Chun, et al. "Functional annotation of rheumatoid arthritis and osteoarthritis associated genes by integrative genome-wide gene expression profiling analysis." *PloS one* 9.2 (2014): e85784.
